# History and Current Status of Evidence-Based Korean Medicine

**DOI:** 10.1155/2014/503867

**Published:** 2014-04-15

**Authors:** Seong-Gyu Ko, Bharat B. Aggarwal, Mingyao Liu, Chang Shik Yin, Dong Hoon Jin, Bo-Hyoung Jang

**Affiliations:** ^1^Laboratory of Clinical Biology and Pharmacogenomics and Center for Clinical Research and Genomics, Kyung Hee University, Seoul 130-701, Republic of Korea; ^2^Department of Experimental Therapeutics, The University of Texas M. D. Anderson Cancer Center, 1901 East Road, Houston, TX 77054, USA; ^3^School of Life Science and Institute of Biomedical Science, East China Normal University, Shanghai 200241, China; ^4^Acupuncture and Meridian Science Research Center, Kyung Hee University, Seoul 130-701, Republic of Korea; ^5^Institute for Innovative Cancer Research, Asan Institute for Life Sciences, Asan Medical Center, University of Ulsan College of Medicine, Seoul 138-736, Republic of Korea; ^6^Office of Health Services Research, National Evidence-Based Healthcare Collaborating Agency, Seoul 110-450, Republic of Korea

The biomedical effects of natural products have been increasingly identified. Korean medicine originates from prehistoric times and shares its origin with Chinese medicine. However, Korean medicine, compared with the Chinese medicine, is not well known relatively. Though Korean medicine and Chinese medicine have a lot in common, Korean medicine has developed its own realm by developing unique approaches and research—“Four-Constitution (Sasang Constitutional) Medicine” as one of the diagnostic tools and Saam acupuncture as one of the treatments. In recent years, Korean medicine expanded its usages, thus, by integrating both traditional and modern approaches. Korean medicine covers many complementary and alternative approaches that are actively validated and developed. These efforts in the field of Korean medicine have been, and will be, an important contribution to the evidence-based complementary and alternative medicine.

This special issue will introduce you to the history and current status of evidence-based Korean medicine with a focus on the recent developments. The following is a brief overview of the articles in this special issue.

Biological activities of a compound or a mixture of herbal medicines in Korean medicine are explored on such conditions as nephrotoxicity, experimental colitis, vasoconstriction, cancer cell growth, multidrug resistant cancer cell growth, neutropenia, obesity and adipogenesis, adipocyte differentiation, cartilage degradation in arthritis, and inflammatory neuronal damage or on such an effect as stimulating osteogenic differentiation of bone marrow mesenchymal stem cells. For the sake of safe and effective use of herbal medicine in Korean medicine, potential interaction has to be cleared. Interaction between herb and herb or between herb and drug was investigated. Traditional rules on combining herbs for synergistic effect were validated. Effects on cytochrome P450 enzyme mediated drug metabolism were investigated in relation to herbal mixture. Randomized controlled trials were reported on the effect of Korean herbal medicine on dysmenorrhea and obesity.

A new real-time measurement system using a wireless inertial measurement unit was developed and reported to be reliable and effective in measuring three-dimensional cervical spine movements. As an effort to explore the diagnostic concept of Korean medicine, conceptual perception on pattern identification was explored with regard to a phlegm pattern. Recent developments on voice-based approach to Four-Constitution Medicine were reviewed. Tinnitus patients treated in Korean medicine clinic were shown to be on sympathetic overactivity with chronic tinnitus more affecting the autonomic indices.

In this special issue, several newly developed treatments are also introduced. The effect of cosmetic acupuncture was explored on facial elasticity in an open-label, single-arm pilot study. The effect of newly developed temporomandibular joint therapy targeting the postural Yin-Yang balance of the whole body was documented in a case series study. Detoxification program involving fasting, fluid administration, acupuncture, and herbal wet wrap dressing was reported to be positive for refractory cases of atopic dermatitis. As an exploration on varied application of herbal medicine, it was found to be safe and effective in hypertensive blood pressure when applied as enema agent. These therapies warrant further study with more rigid study design.

*Seong-Gyu  Ko* *Seong-Gyu  Ko* *Bharat  B.  Aggarwal* *Bharat  B.  Aggarwal* *Mingyao  Liu* *Mingyao  Liu* *Chang  Shik  Yin* *Chang  Shik  Yin* *Dong  Hoon  Jin* *Dong  Hoon  Jin* *Bo-Hyoung  Jang* *Bo-Hyoung  Jang*

